# The Newer Treatments of Syphilis

**Published:** 1913-07-26

**Authors:** 


### THE NEWER TREATMENTS OF SYPHILIS.

### Some Possible Sociological Effects.

Whether it came with Columbus' crews from the West or was brought by Crusaders from the East, syphilis, in Europe, has decimated armies and brought to naught the schemes of princes. It has caused and is causing more physical and mental inefficiency than all the plagues that have smitten the races of the earth. Those only whose daily work is amongst the out-patients of great hospitals or the in-patients of our asylums and infirmaries have a glimmering of the amount of the misery that results from the late manifestations of this appalling disease.

For years past, as those who will study the official reports and the articles in the *Journal of the Royal Army Medical Corps* may learn, the Corps has done a vast amount of work, the end of which has been to systematise and make perfect a treatment for syphilis. It was but to be expected, therefore, that, when salvarsan was discovered, it was eagerly seized upon by the Army doctors as a likely instrument to the end in view. A series of experiments was commenced to ascertain the power and the possibilities of the new drug. These researches are not yet ended, but a method of treatment has been evolved to a point so far beyond the experimental stages that the medical authorities have considered the time ripe for issue to the officers of the Royal Army Medical Corps of a pamphlet headed "Instructions Regarding the Methods to be Adopted for the Diagnosis and Treatment of Cases of Syphilis." The few unpretentious sheets of the pamphlet set forth, in the clearest terms, a method of treatment which combines the intra-muscular injection of mercury with the intravenous injection of salvarsan, and which evidently appears to the men working in the venereal hospital at Rochester Row to be that which has in it the most promise of real success and permanent cure. It is not proposed here to enter into the details set forth in the instructions, but to point out the vista that is opened up by the fact that such instructions have been issued by authority.

Perusal of the measures to be taken to ascertain if a cure has resulted, for instance, leaves the impression that there is a well-founded hope that permanent cure may result from a course of treatment which extends over a period of less than three months. If this should prove to be a usual result, the effect on the health of the community will be marked beyond present conception—that vast agglomeration of gummata, chronic ulcers, and other tertiary syphilitic manifestations that form so large a proportion of the clinical material collected in the out-patient departments of our hospitals will disappear.

Lunatic asylums will seldom have to care for sufferers from general paralysis, and will lose much, perhaps most, of their supply of non-hereditary cases of insanity. With a shortened period of contagiousness there will be less opportunity for the infected to infect in their turn. This must result in a decrease in the number of fresh cases. That, again, will be one factor the more lessening the incidence of the disease. The number of the infected will be diminished to a much smaller percentage of the population. With this reduction one of the objections to making syphilis a notifiable disease will disappear, or be over-ruled from lack of numbers to support it; the objection born of the fear of a considerable proportion of the community that notification will result in publicity being given to its secret sins. Then measures will be taken to isolate the infected till they cease to be contagious, and, in time, syphilis of native origin may be as rare in the British Isles as leprosy. None can calculate in advance the gain in communal health and national efficiency that such a result would bring.

In this connection we would draw attention to the very able plea, advanced with a dignity and moderation which it would be hard to over-praise, by Sir Malcolm Morris for a Royal Commission upon Venereal Disease, published in the *Lancet* a month ago; and to the correspondence which has ensued in the columns of the same journal. There are, of course, many people who hold strong views upon the methods which should be used by society for the prevention and the extermination of these diseases; and others in plenty, just as earnest and just as intelligent, who cling tenaciously to views exactly the opposite. Both sides would have their opportunities of expounding their arguments to a Commission such as that which Sir Malcolm Morris proposes; and there is no getting away from the fact that the researches of the last four or five years have entirely altered many of the factors in the problem of the control of venereal infections.

